# Morphological and Phylogenetic Analyses Reveal Three New Species of *Distoseptispora* (*Distoseptisporaceae*, *Distoseptisporales*) from Yunnan, China

**DOI:** 10.3390/jof9040470

**Published:** 2023-04-14

**Authors:** Jingwen Liu, Yafen Hu, Xingxing Luo, Zhaohuan Xu, Rafael F. Castañeda-Ruíz, Jiwen Xia, Xiuguo Zhang, Lianhu Zhang, Ruqiang Cui, Jian Ma

**Affiliations:** 1 College of Agronomy, Jiangxi Agricultural University, Nanchang 330045, China; 2 Instituto de Investigaciones de Sanidad Vegetal, Calle 110 No. 514 e/5ta B y 5ta F, Playa, La Habana 11600, Cuba; 3 Shandong Provincial Key Laboratory for Biology of Vegetable Diseases and Insect Pests, College of Plant Protection, Shandong Agricultural University, Tai’an 271018, China

**Keywords:** asexual ascomycetes, hyphomycetes, new taxa, phylogeny, *Sordariomycetes*, taxonomy

## Abstract

Three new species of *Distoseptispora*, viz. *D*. *mengsongensis*, *D*. *nabanheensis*, and *D*. *sinensis*, are described and illustrated from specimens collected on dead branches of unidentified plants in Yunnan Province, China. Phylogenetic analyses of LSU, ITS, and *TEF1* sequence data, using maximum-likelihood (ML) and Bayesian inference (BI), reveal the taxonomic placement of *D*. *mengsongensis*, *D*. *nabanheensis*, and *D*. *sinensis* within *Distoseptispora*. Both morphological observations and molecular phylogenetic analyses supported *D*. *mengsongensis*, *D*. *nabanheensis*, and *D*. *sinensis* as three new taxa. To extend our knowledge of the diversity of *Distoseptispora*-like taxa, a list of recognized species of *Distoseptispora* with major morphological features, habitat, host, and locality is also provided.

## 1. Introduction

The genus *Distoseptispora* K.D. Hyde, McKenzie & Maharachch was established by Su et al. [1] with *D. fluminicola* McKenzie, Hong Y. Su, Z.L. Luo & K.D. Hyde as the type species, and was mainly characterized by being macronematous, septate, unbranched, smooth, olivaceous to brown conidiophores with monoblastic, integrated, terminal, determinate, cylindrical conidiogenous cells that produce acrogenous, solitary, distoseptate conidia. Subsequently, the phylogenetic analyses motivated the inclusion of taxa with euseptate conidia (e.g., *D. guttulata* J. Yang & K.D. Hyde and *D. suoluoensis* J. Yang, Maharachch. & K.D. Hyde) [2] and polyblastic conidiogenous cells (e.g., *D*. *palmarum* S.N. Zhang, K.D. Hyde & J.K. Liu) [3]. To date, 61 epithets for *Distoseptispora* are listed in Index Fungorum [4]. Monkai et al. [5] provided a synopsis of relevant morphological features that distinguish 29 *Distoseptispora* species. Zhai et al. [6] subsequently published an expanded synopsis that includes additional nine species following the same format as Monkai et al. [5], but ignored the species *D*. *submersa* Z.L. Luo, K.D. Hyde & H.Y. Su, which was regarded as the synonym of *D*. *tectonae* Doilom & K.D. Hyde by Dong et al. [7]. Thus, *Distoseptispora* currently contains 60 taxa, 38 of which were found in China [1,2,3,6,8,9,10,11,12,13,14,15,16,17,18,19,20,21].

*Distoseptispora* is one of the *Sporidesmium*-like genera with high morphological similarity to the *Sporidesmium* Link and *Ellisembia* Subram. *Distoseptispora* species with eu- or distoseptate conidia covering the criteria of *Ellisembia* and *Sporidesmium*. Accordingly, *Distoseptispora* species cannot be classified based on morphology alone, as phylogenetic analysis showed that these genera are not closely related [1,2,22]. *Distoseptispora* also appears similar in conidial ontogeny to *Aquapteridospora* Jiao Yang, K.D. Hyde & Maharachch., but the latter has terminal and intercalary conidiogenous cells with circular scars, and produces 3-euseptate conidia [23,24]. Additionally, *Distoseptispora* formed a sister clade to *Aquapteridospora*, and is well separated with high support in phylogenetic trees [24]. *Distoseptispora* and *Aquapteridospora* belonged to the order *Distoseptisporales* Z.L. Luo, K.D. Hyde & H.Y. Su, but are now, respectively, treated in the families *Distoseptisporaceae* K.D. Hyde & McKenzie and *Aquapteridosporaceae* K.D. Hyde & Hongsanan [24,25].

*Distoseptispora* species are primarily found as saprobes on submerged wood, dead branches, culms, or leaves in freshwater or terrestrial habitats except for *D*. *caricis* Crous and *D. palmarum* S.N. Zhang, K.D. Hyde & J.K. Liu occurring on the leaves of *Carex* sp. and rachis of *Cocos nucifera* [3,6,26]. Species of the genus decompose lignocellulose in wood [22,27,28], but their ecological functions, geographical distribution, alpha-taxonomy, and teleomorph relationships are poorly known. During our continuing survey (2007–2022) of saprophytic microfungi from the forest ecosystems of southwest China, several *Sporidesmium*-like taxa were isolated on dead branches of unidentified perennial dicotyledonous plants from terrestrial habitats in Yunnan Province, China. Using multi-gene loci of LSU, ITS, and *TEF1* sequence data, the systematic placement of these isolates represented several *Distoseptispora* species. Based on morphological characteristics and multi-locus phylogenetic analysis, three new species of *Distoseptispora*, viz. *D*. *mengsongensis*, *D*. *nabanheensis*, and *D*. *sinensis*, are proposed and described in this paper.

## 2. Materials and Methods

### 2.1. Sample Collection, Isolation, and Morphological Examination

Samples of decomposing wood and bark were collected from the forest floor in Yunnan Province, China, and brought them back to the laboratory in Ziploc™ bags. Samples were treated following the methods described by Ma et al. [29]. Colonies on the surface of dead branches were examined and visually observed with a stereomicroscope (Motic SMZ-168, Xiamen, China) from low (7.5 times) to high (50 times) magnification. Fresh colonies were picked with sterile needles at a stereomicroscope magnification of 50 times, placed on a slide with a drop of lactic acid–phenol solution (lactic acid, phenol, glycerin, sterile water; 1:1:2:1, respectively), then placed under an Olympus BX 53 light microscope fitted with an Olympus DP 27 digital camera (Olympus Optical Co., Ltd., Tokyo, Japan) for microscopic morphological characterization. The tip of a sterile toothpick dipped in sterile water was used to pick conidia from the specimen; the conidia were then streaked on the surface of potato dextrose agar (PDA; 20% potato + 2% dextrose + 2% agar, *w/v*) and incubated at 25 °C overnight. Single-spore isolations were made on potato dextrose agar (PDA) following Goh [30]. Colony colors were assessed according to the charts of Rayner [31]. All fungal strains were stored in 10% sterilized glycerin at 4 °C for further studies. The studied specimens and cultures were deposited in the Herbarium of Jiangxi Agricultural University, Plant Pathology, Nanchang, China (HJAUP).

### 2.2. DNA Extraction, PCR Amplification, and Sequencing

Fungal hyphae (500 mg) were scraped from the surface of colonies growing on PDA plates, transferred to 2 mL safe-lock microtubes, and ground with liquid nitrogen. DNA was extracted using the Solarbio Fungal Genomic DNA Extraction Kit (Beijing Solarbio Science & Technology Co., Ltd., Beijing, China) according to the manufacturer’s instructions. Primer sets were used for the amplification of LSU and ITS, and *TEF1*: ITS5/ITS4 [32], 28S1-F/28S3-R [8], and EF1-983F/EF1-2218R [33]. The final volume of the PCR reaction was 25 μL, containing 1 μL of DNA template, 1 μL of each forward and reward primer, 12.5 μL of 2 × Power Taq PCR MasterMix, and 9.5 μL of double-distilled water (ddH_2_O). The PCR thermal cycling conditions of ITS and LSU were initialized at 94 °C for 3 min, followed by 35 cycles of denaturation at 94 °C for 30 s, annealing at 55 °C for 50 s, elongation at 72 °C for 1 min, a final extension at 72 °C for 10 min, and finally kept at 4 °C. *TEF1* was initialized at 94 °C for 3 min, followed by 35 cycles of denaturation at 94 °C for 30 s, annealing at 52 °C for 30 s, elongation at 72 °C for 1 min, a final extension at 72 °C for 10 min, and finally kept at 4 °C. The PCR products were checked on 1% agarose gel electrophoresis stained with ethidium bromide. Purification and DNA sequencing were carried out at Beijing Tsingke Biotechnology Co., Ltd., Beijing, China.

### 2.3. Sequence Alignment and Phylogenetic Analyses

Sequences, including those obtained from GenBank (Table 1), were initially aligned using MAFFTv.7 [34] on the online server (http://maffTh.cbrc.jp/alignment/server/, accessed on 1 February 2023) and optimized manually when needed. The LSU, ITS, and *TEF1* sequence data were concatenated by using Phylosuite software v1.2.1 [35], and absent sequence data in the alignments were treated with the question mark and “-” as missing data. The phylogenetic tree was constructed using Phylosuite software v1.2.1 [35] based on the combined data of LSU, ITS, and *TEF1* sequence (Supplementary Materials). The concatenated aligned dataset was analyzed separately using maximum likelihood (ML) and Bayesian inference (BI). Maximum-likelihood phylogenies were inferred using IQ-TREE [36] under edge-linked partition model for 10,000 ultrafast [37] bootstraps. The final tree was selected among suboptimal trees from each run by comparing the likelihood scores using the TNe+I+G4 for ITS, TNe+R3 for LSU, and TN+F+I+G4 for *TEF1* substitution model. Bayesian inference phylogenies were inferred using MrBayes 3.2.6 [38] under partition model (2 parallel runs, 2,000,000 generations), in which the initial 25% of sampled data were discarded by burning. The best-fit model was GTR+F+I+G4 for ITS+*TEF1*, and GTR+F+I+G4 for LSU. ModelFinder was used to select the best-fit partition model (edge-linked) using BIC criterion [39]. The trees were viewed in FigTree v. 1.4.4 (http://tree.bio.ed.ac.uk/software/figtree, accessed on 1 February 2023), and further edited in Adobe Illustrator 2021. Sequences generated in this study were deposited in GenBank (Table 1).

## 3. Results

### 3.1. Molecular Phylogeny

**Figure 1:**
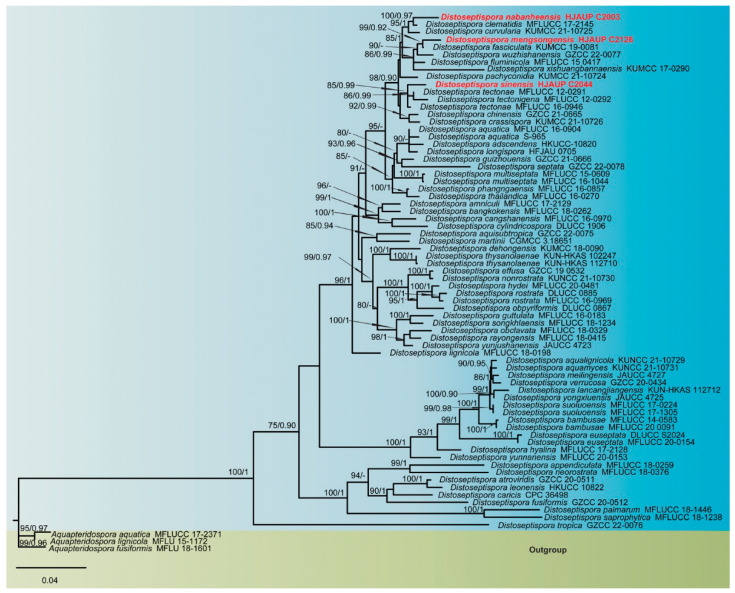
Phylogenetic tree inferred from maximum-likelihood and Bayesian inference analyses based on concatenated LSU, ITS, and *TEF1* sequences. Significant MLBS/BPP support values above 75% and 0.90 are indicated at the nodes. The new isolates of this study are shown in red. The tree is rooted to *Aquapteridospora aquatica* (MFLUCC 17-2371), *A. fusiformis* (MFLU 18-1601), and *A. lignicola* (MFLU 15-1172).

The combined sequence alignment comprised 72 strains representing 64 species (Table 1), 2248 total characters (ITS:1–493, LSU:494–1329, *TEF1*:1330–2248), including 891 distinct patterns, 537 parsimony-informative, 275 singleton sites, and 1436 constant sites), and used *Aquapteridospora aquatica* (MFLUCC 17-2371), *A. fusiformis* (MFLUCC 18-1606) and *A. lignicola* (MFLUCC 15-0377) as outgroup. Maximum-likelihood (ML) and Bayesian inference (BI) analyses of the combined dataset resulted in phylogenetic reconstructions with largely similar topologies, and the best-scoring ML tree (lnL = −16,099.937) is shown in Figure 1. Maximum-likelihood bootstrap support (MLBS) values above 75% and Bayesian posterior probabilities (BPP) greater than 0.90 are given above the nodes. Our three isolates in this lineage formed distinct clades with good support value, and can be recognized as three new phylogenetic species, *Distoseptispora mengsongensis*, *D*. *nabanheensis*, and *D*. *sinensis*. Phylogenetic analyses suggested sister group relatedness of *D*. *nabanheensis* (HJAUP C2003) and *D*. *clematidis* (MFLUCC 17-2145) (MLBS/BPP = 100/0.97); *D*. *mengsongensis* (HJAUP C2126) and *D*. *fasciculata* (KUMCC 19-0081) (MLBS/BPP = 99/0.92); and *D*. *sinensis* (HJAUP C2044), *D*. *tectonae* (MFLUCC 12-0291, MFLUCC 16-0946), and *D. tectonigena* (MFLUCC 12-0292) (MLBS/BPP = 85/0.99).

### 3.2. Taxonomy

**Figure 2:**
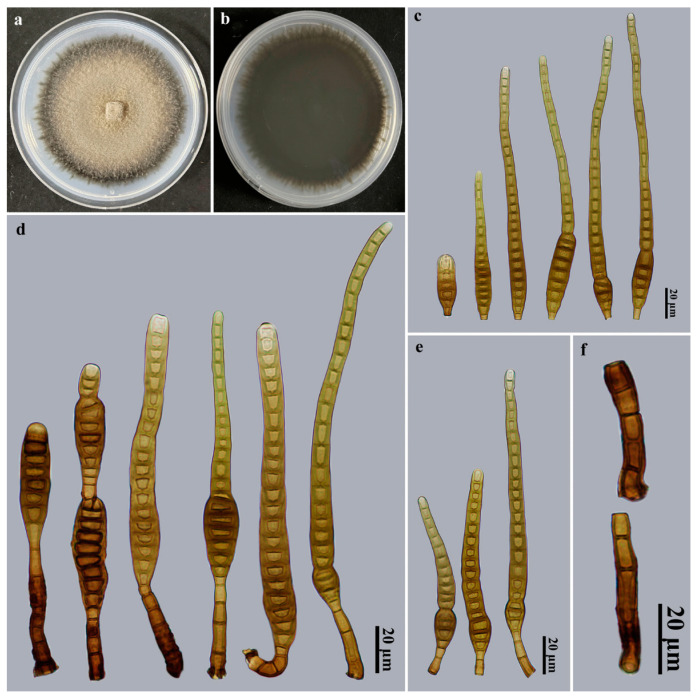
*Distoseptispora mengsongensis* (HJAUP M2126, holotype). (**a**) Surface of colony after 2 weeks on PDA; (**b**) reverse of colony after 2 weeks on PDA; (**c**) Conidia; (**d**,**e**) Conidiophores, conidiogenous cells, and conidia; (**f**) Conidiophores.

*Distoseptispora mengsongensis* Jing W. Liu, X.G. Zhang & Jian Ma, sp. nov., Figure 2.

Index Fungorum number: IF900033.

Etymology: In reference to the locality, Mengsong Township, where the fungus was collected.

Holotype: HJAUP M2126.

Description: Saprobic on dead branches in terrestrial habitats. *Teleomorph*: Undetermined. *Anamorph* (Figure 2): Hyphomycetes. *Colonies* on natural substratum effuse, brown, hairy. *Mycelium* is superficial and immersed, composed of branched, septate, pale brown to brown, smooth-walled hyphae. *Conidiophores* macronematous, mononematous, cylindrical, 1–5-septate, erect, unbranched, smooth, straight or slightly flexuous, brown to dark brown, 17–54 × 4.5–7 μm ($\bar{x}$ = 33.5 × 5.5 μm, *n* = 20). *Conidiogenous cells* monoblastic, integrated, terminal, cylindrical, flat at the conidiogenous loco, determinate, pale brown to brown, smooth, 8.5–14.2 × 3–5.7 μm ($\bar{x}$ = 11.3 × 4.1 μm, *n* = 20). *Conidial secession* schizolytic. *Conidia* acrogenous, solitary, obclavate, 15–31-distoseptate, sometimes constricted at the septa, especially in proximal parts, straight or slightly curved, smooth, brown to dark brown, sometimes with percurrent regeneration forming a secondary conidium from the conidial apex, 86–200 × 6–13 μm ($\bar{x}$ = 141 × 9.7 μm, *n* = 25), base truncate and 3–5.5 μm wide, apex rounded, 2.9–8.6 μm wide.

Culture characteristics: Colony on PDA reached 81–86 mm diam. after 2 weeks in an incubator under dark conditions at 25 °C, irregularly rounded, surface velvety, with dense, brown mycelium, margin entire, dark brown to black; the reverse is black.

Material examined: China, Yunnan Province, Xishuangbanna Dai Autonomous Prefecture, Menghai County, Mengsong Township, on dead branches of an unidentified broadleaf tree, 12 July 2021, J.W. Liu, HJAUP M2126 (**holotype**), ex-type culture permanently preserved in a metabolically inactive state, HJAUP C2126.

Notes: Phylogenetic analyses showed that *D. mengsongensis* (HJAUP C2126) clusters with *D*. *fasciculata* (KUMCC 19-0081). BLASTn analysis of *D*. *mengsongensis* (HJAUP C2126) and *D*. *fasciculata* (KUMCC 19-0081) showed 99% identity (544/551, no gaps) using ITS, 99% identity (565/567, two gaps) using LSU, and 100% identity (927/927, no gaps) using *TEF1*. Moreover, *D. mengsongensis* morphologically differs from *D*. *fasciculata* W. Dong, H. Zhang & K.D. Hyde, which occurs in freshwater habitats and has smaller conidiophores (12–16 × 5–6 μm) and wider conidia (10–16.5 μm wide) [7]. *Distoseptispora mengsongensis* is morphologically similar to *D*. *xishuangbannaensis* Tibpromma & K.D. Hyde, but the latter differs by occurring on dead leaf sheath and not on wood, and further differs by its shorter and narrower conidiophores (12–17 × 2–5 μm) and bigger conidia (160–305 × 8–15 μm) with up to 40 distosepta [10].

**Figure 3:**
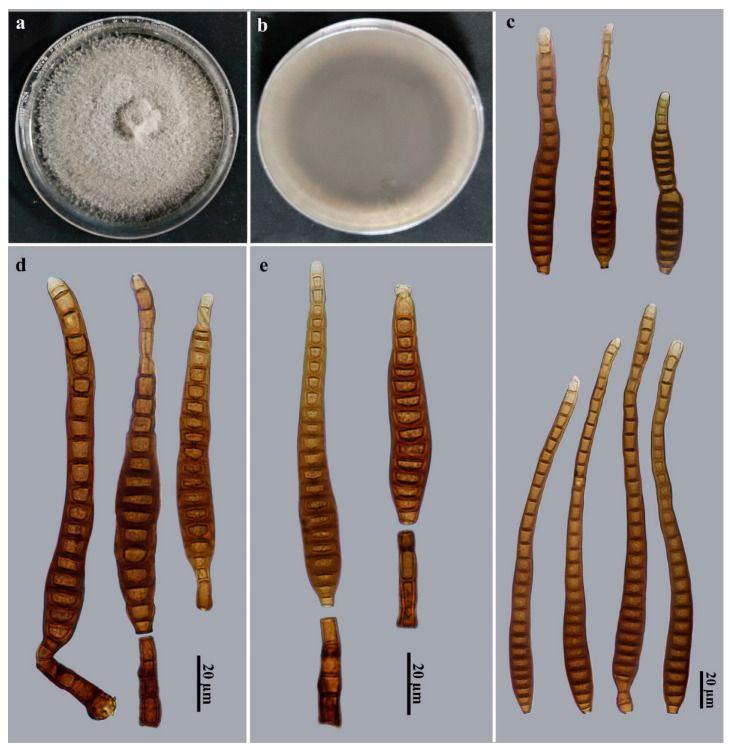
*Distoseptispora nabanheensis* (HJAUP M2003, holotype). (**a**) Surface of colony after 2 weeks on PDA; (**b**) reverse of colony after 2 weeks on PDA; (**c**) Conidia; (**d**,**e**) Conidiophores, conidiogenous cells, and conidia.

*Distoseptispora nabanheensis* Jing W. Liu, X.G. Zhang & Jian Ma, sp. nov., Figure 3.

Index Fungorum number: IF900054.

Etymology: In reference to the locality, Nabanhe Nature Reserve, in which the fungus was collected.

Holotype: HJAUP M2003.

Description: Saprobic on dead branches in terrestrial habitats. *Teleomorph*: Undetermined. *Anamorph* (Figure 3): Hyphomycetes. *Colonies* on natural substratum effuse, brown, hairy. *Mycelium* is superficial and immersed, composed of branched, septate, pale brown to brown, smooth-walled hyphae. *Conidiophores* macronematous, mononematous, cylindrical, 3–8-septate, erect, unbranched, solitary, smooth, straight or slightly flexuous, brown to dark brown, 29–42 × 8–10 μm ($\bar{x}$ = 37.5 × 9.2 μm, *n* = 9). *Conidiogenous cells* monoblastic, integrated, terminal, cylindrical, flat at the conidiogenous loco, determinate, brown to pale brown, smooth, 4–6 × 4–5 μm ($\bar{x}$ = 5.2 × 4.6 μm, *n* = 9). *Conidial secession* schizolytic. *Conidia* acrogenous, solitary, obclavate, 18–31-distoseptate, slightly constricted at the septa, smooth, brown to dark brown, 102–214.5 × (7–)11–14.5 μm ($\bar{x}$ = 171 × 11.3 μm, *n* = 20), base truncate and 3.5–7.5 μm wide, apex rounded, 3.2–8 μm wide.

Culture characteristics: Colony on PDA reached 83–88 mm diam. after 2 weeks in an incubator under dark conditions at 25 °C, circular, surface velvety, with dense, gray mycelium on the surface along the entire margin; the reverse is dark brown to black.

Material examined: China, Yunnan Province, Xishuangbanna Dai Autonomous Prefecture, the Nabanhe National Nature Reserve, on dead branches of an unidentified broadleaf tree, 12 July 2021, J.W. Liu, HJAUP M2003 (**holotype**), ex-type culture permanently preserved in a metabolically inactive state, HJAUP C2003.

Notes: Phylogenetic analyses showed that *D. nabanheensis* (HJAUP C2003) clusters with *D*. *clematidis* (MFLUCC 17-2145). BLASTn analysis of *D*. *nabanheensis* (HJAUP C2003) and *D*. *clematidis* (MFLUCC 17-2145) shows 99% identity (531/533, no gaps) using ITS, 99% identity (471/477, two gaps) using LSU. Moreover, *D. nabanheensis* morphologically differs from *D*. *clematidis* Phukhams., M.V. de Bult & K.D. Hyde, which has smaller conidiophores (22–40 × 4–10 μm) and wider conidia (12–20 μm wide) with 28–35 distosepta [41]. *Distoseptispora nabanheensis* is morphologically similar to *D*. *chinensis* X. Tang, Jayaward., J.C. Kang & K.D. Hyde, and *D*. *tectonigena* Doilom & K.D. Hyde, but *D*. *chinensis* lives in freshwater habitats and differs by its narrower conidiophores (5.5–9 μm wide) and bigger conidia (81–283 × 10–19 μm), with up to 40 distosepta [12]; *D*. *tectonigena* differs by its longer conidiophores (up to 110 μm long) and longer conidia (83–360 μm long), with 20–46 distosepta [42].

**Figure 4:**
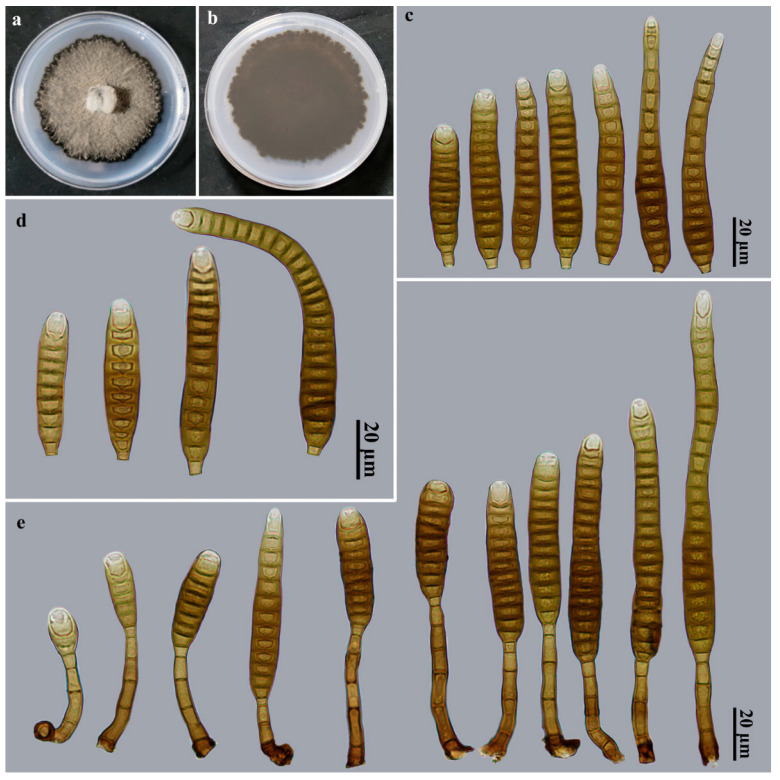
*Distoseptispora sinensis* (HJAUP M2044, holotype). (**a**) Surface of colony after 2 weeks on PDA; (**b**) reverse of colony after 2 weeks on PDA; (**c**,**d**) Conidia; (**e**) Conidiophores, conidiogenous cells, and conidia.

*Distoseptispora sinensis* Jing W. Liu, X.G. Zhang & Jian Ma, sp. nov., Figure 4.

Index Fungorum number: IF900055.

Etymology: In reference to the country “China” in which the fungus was collected.

Holotype: HJAUP M2044.

Description: Saprobic on dead branches in terrestrial habitats. *Teleomorph*: Undetermined. *Anamorph*: Hyphomycetes. *Colonies* on natural substratum effuse, brown, hairy. *Mycelium* is superficial and immersed, composed of branched, septate, pale brown to brown, smooth-walled hyphae. *Conidiophores* macronematous, mononematous, cylindrical, 2–5-septate, erect, unbranched, solitary, smooth, straight or slightly flexuous, brown to dark brown, 23.5–56.5 × 3.5–7 μm ($\bar{x}$ = 39.6 × 5 μm, *n* = 20). *Conidiogenous cells* monoblastic, integrated, terminal, cylindrical, flat at the conidiogenous loco, determinate, pale brown, smooth, 6.5–10 × 3.3–3.6 μm ($\bar{x}$ = 8.3 × 3.5 μm, *n* = 20). *Conidial secession* schizolytic. *Conidia* acrogenous, solitary, obclavate, straight or slightly curved, smooth, 10–25-distoseptate, brown to dark brown, apical cell paler, 40–107(–137) × 10–12 μm ($\bar{x}$ = 78.8 × 10.5 μm, *n* = 30), base truncate and 3–3.5 μm wide, apex rounded, 3.5–10 μm wide.

Culture characteristics: Colony on PDA reaching 64–69 mm diam. after 2 weeks in an incubator under dark conditions at 25 °C, irregularly rounded, surface velvety, with dense, gray mycelium, black at the entire margin; the reverse is dark brown to black.

Material examined: China, Yunnan Province, Xishuangbanna Dai Autonomous Prefecture, Jinghong City, Gasa Township, on dead branches of an unidentified broadleaf tree, 12 July 2021, J.W. Liu, HJAUP M2044 (**holotype**), ex-type culture permanently preserved in a metabolically inactive state, HJAUP C2044.

Notes: Phylogenetic analyses showed that *D. sinensis* (HJAUP C2044) clusters with *D*. *tectonae* (MFLUCC 12–0291^T^, MFLUCC 16–0946) and *D. tectonigena* (MFLUCC 12–0292). BLASTn analysis of *D*. *sinensis* (HJAUP C2044) and *D*. *tectonae* (MFLUCC 12-0291^T^) shows 98% identity (559/569, five gaps) using ITS, 98% identity (574/583, five gaps) using LSU, and 99% identity (926/930, no gaps) using *TEF1*; BLASTn analysis of *D. sinensis* (HJAUP C2044) and *D. tectonigena* (MFLUCC 12–0292) shows 96% identity (549/570, seven gaps) using ITS and 99% identity (575/583, five gaps) using LSU. Moreover, *D*. *sinensis* morphologically differs from *D*. *tectonae*, which has smaller conidiophores (up to 40 × 4–6 μm) and larger conidia (90–170 × 11–16 μm) with 20–28 distosepta [42], as well as from *D. tectonigena*, which has larger conidiophores (up to 110 × 5–11 μm) and larger conidia (83–360 × 10–13 μm), with 20–46 distosepta [42].

## 4. Discussion

*Sporidesmium*-like taxa have undergone convergent evolution, and the morphological characteristics used to delimit *Sporidesmium*-like genera are shown to be insignificant in a phylogenetic context. Responding to the heterogeneity of *Sporidesmium*, the genus *Distoseptispora* was introduced by Su et al. [1] based on multi-locus phylogenies together with morphology. In recent years, the number of *Distoseptispora* species steadily increased and currently reached 63 species, including *D*. *mengsongensis*, *D*. *nabanheensis*, and *D*. *sinensis*. In the phylogenetic tree (Figure 1), some *Distoseptispora* species form sister clades, but they show different morphological characteristics, such as how *D. mengsongensis* and *D. fasciculata* are clustered, but the conidia of *D. mengsongensis* are obclavate, constricted at the septa, especially in proximal parts, sometimes with percurrent regeneration forming a secondary conidium from the conidial apex, with an average conidial length/width ratio of 14.54, while the conidia of *D. fasciculata* are subcylindrical to obclavate, with an average conidial length/width ratio of 8.44. *Distoseptispora nabanheensis* and *D. clematidis* have a close phylogenetic relationship, but *D. nabanheensis* has obclavate, slightly constricted at the septa, brown to dark brown conidia, with an average conidial length/width ratio of 15.13, while *D. clematidis* has oblong, obclavate, cylindrical or rostrate, brown with green tinge conidia, with an average conidial length/width ratio of 10.29. *Distoseptispora sinensis*, *D. tectonigena*, and *D. tectonae* have different morphology of conidiophores and conidia, and comparisons of nucleotides between *D. tectonae* (MFLUCC 12-0291^T^) and our isolate (HJAUP C2044) showed 10 and 9 (2%, including five gaps) nucleotide differences in ITS and LSU regions, respectively; *D. tectonigena* (MFLUCC 12–0292^T^) and our isolate (HJAUP C2044) showed 21 and 8 (4%, including seven gaps; 1.2%, including five gaps) nucleotide differences in ITS and LSU regions, respectively. Considering this scenario, additional molecular data and morphological characteristics are required for verification and expansion.

To date, all *Distoseptispora* species were identified by morphological and phylogenetic analyses, which led to a better evaluation of their phylogenetic relationships and taxonomic placements. However, studies conducted on *Distoseptispora* have no universally accepted standards with respect to barcode selection for phylogenetic analyses. For instance, Su et al. [1] established the genus *Distoseptispora*, but the initial species had only LSU sequences. Tibpromma et al. [10] introduced *D. thailandica* Tibpromma & K.D. Hyde and *D. xishuangbannaensis* using LSU and ITS. Monkai et al. [5] introduced *D. hydei* Monkai & Phookamsak using LSU, ITS, and *RPB2*. Hyde et al. [12] introduced *D. chinensis* X. Tang, Jayaward., J.C. Kang & K.D. Hyde and *D. guizhouensis* X. Tang, Jayaward., J.C. Kang & K.D. Hyde using ITS, LSU, SSU, *TEF1*, and *RPB2*. Zhai et al. [6] introduced *D. meilingensis* Z.J. Zhai & D.M. Hu, *D. yongxiuensis* Z.J. Zhai & D.M. Hu, and *D. yunjushanensis* Z.J. Zhai & D.M. Hu using ITS, LSU, SSU, and *TEF1*. Ma et al. [20] and Zhang et al. [21] introduced 10 *Distoseptispora* species using LSU, ITS, *TEF1*, and *RPB2*. The recent studies also indicated that the use of only LSU and ITS sequences might be problematic in resolving the phylogeny of *Distoseptisporaceae*, as *RPB2* and/or *TEF1* usually increased phylogenetic resolution significantly. In our study, we conducted phylogenetic analyses based on combined LSU, ITS, and *TEF1* sequences, and obtained good phylogenetic support. Our three species, viz. *D*. *mengsongensis*, *D*. *nabanheensis*, and *D*. *sinensis*, are considerably distinct from all other described *Distoseptispora* species by morphological characteristics and multi-locus phylogenetic analysis, and thus we are convinced that the newly introduced species are new to science.

Studies conducted to date on *Distoseptispora* are mainly focused on their alpha-taxonomy, and most species of this genus are known from dead parts of plants as saprobic fungi in aquatic and terrestrial habitats [5,11,12,17,19,20,21,43], except *D. caricis* and *D. palmarum* which are reported, respectively, on leaves of *Carex* sp. and rachis of *Cocos nucifera* on [3,6,26], whereas we have little attention on their roles in ecosystem function. They total 63 valid species (Table 2 and Table 3), 44 of which are from freshwater, and 19 are from terrestrial habitats. Most *Distoseptispora* species are described based on their anamorph alone, and only two species, *D. hyalina* and *D. licualae* (Table 3), are reported as sexual morphs based on molecular DNA data, but the connection of teleomorph and anamorph has not been proved by pure culture or sequence data. The genus *Distoseptispora* has mainly been reported in China (41 species) and Thailand (22 species) [4], and only a small amount of published information is recorded in other regions (e.g., Hungary, Malaysia, Puerto Rico, Sierra Leone) [4,6,44]. Thus, it is unclear whether it has a close relationship with geographic regions, but we expect that large-scale surveys of *Distoseptispora* in aquatic and terrestrial habitats within different geographic regions, ecological environments, and climatic conditions are needed. This will contribute to a comprehensive knowledge of the species diversity of this genus, and further evaluate their phylogenetic relationships and taxonomic placements by molecular methods.

## Figures and Tables

**Table 1:** List of *Distoseptispora* species and GenBank accessions used in the phylogenetic analyses. New sequences are in bold.

Taxon	Voucher/Strain Number	GenBank Accession Numbers			References
		LSU	ITS	TEF1	
*Aquapteridospora aquatica*	MFLUCC 17-2371^T^	MW287767	MW286493	—	[7]
*A. fusiformis*	MFLUCC 18-1606^T^	MK849798	MK828652	MN194056	[22]
*A. lignicola*	MFLUCC 15-0377^T^	KU221018	—	—	[23]
*Distoseptispora adscendens*	HKUCC 10820	DQ408561	—	—	[40]
*D. amniculi*	MFLUCC 17-2129^T^	MZ868761	MZ868770	—	[11]
*D. appendiculata*	MFLUCC 18-0259^T^	MN163023	MN163009	MN174866	[22]
*D. aqualignicola*	KUNCC 21-10729^T^	ON400845	OK341186	OP413480	[21]
*D. aquamyces*	KUNCC 21-10731^T^	OK341199	OK341187	OP413482	[21]
*D. aquatica*	MFLUCC 16-0904	MK849794	MK828649	—	[22]
*D. aquatica*	S-965	MK849792	MK828647	MN194051	[22]
*D. aquisubtropica*	GZCC 22-0075^T^	ON527941	ON527933	ON533677	[20]
*D. atroviridis*	GZCC 20-0511^T^	MZ868763	MZ868772	MZ892978	[11]
*D. bambusae*	MFLUCC 20-0091^T^	MT232718	MT232713	MT232880	[15]
*D. bambusae*	MFLUCC 14-0583	MT232717	MT232712	—	[15]
*D. bangkokensis*	MFLUCC 18-0262^T^	MZ518206	MZ518205	—	[17]
*D. cangshanensis*	MFLUCC 16-0970^T^	MG979761	MG979754	MG988419	[9]
*D. caricis*	CPC 36498^T^	MN567632	NR_166325	—	[26]
*D. chinensis*	GZCC 21-0665^T^	MZ474867	MZ474871	MZ501609	[12]
*D. clematidis*	MFLUCC 17-2145^T^	MT214617	MT310661	—	[41]
*D. crassispora*	KUMCC 21-10726^T^	OK341196	OK310698	OP413479	[21]
*D. curvularia*	KUMCC 21-10725^T^	OK341195	OK310697	OP413478	[21]
*D. cylindricospora*	DLUCC 1906^T^	OK513523	OK491122	OK524220	[19]
*D. dehongensis*	KUMCC 18-0090^T^	MK079662	MK085061	MK087659	[3]
*D. effusa*	GZCC 19-0532^T^	MZ227224	MW133916	MZ206156	[11]
*D. euseptata*	MFLUCC 20–0154^T^	MW081544	MW081539	—	[16]
*D. euseptata*	DLUCC S2024	MW081545	MW081540	MW084994	[16]
*D. fasciculata*	KUMCC 19-0081^T^	MW287775	MW286501	MW396656	[7]
*D. fluminicola*	MFLUCC 15-0417^T^	KU376270	MF077553	—	[9]
*D. fusiformis*	GZCC 20-0512^T^	MZ868764	MZ868773	MZ892979	[11]
*D. guizhounesis*	GZCC 21-0666^T^	MZ474869	MZ474868	MZ501610	[12]
*D. guttulata*	MFLUCC 16-0183^T^	MF077554	MF077543	MF135651	[2]
*D. hyalina*	MFLUCC 17-2128^T^	MZ868760	MZ868769	MZ892976	[11]
*D. hydei*	MFLUCC 20-0481^T^	MT742830	MT734661	—	[5]
*D. lancangjiangensis*	KUN-HKAS 112712^T^	MW879522	MW723055	—	[17]
*D. leonensis*	HKUCC 10822^T^	DQ408566	—	—	[40]
*D. lignicola*	MFLUCC 18-0198^T^	MK849797	MK828651	—	[22]
*D. longispora*	HFJAU 0705^T^	MH555357	MH555359	—	[14]
*D. martinii*	CGMCC 3.18651^T^	KX033566	KU999975	—	[8]
*D. meilingensis*	JAUCC 4727^T^	OK562396	OK562390	OK562408	[6]
***D. mengsongensis***	**HJAUP C2126^T^**	**OP787874**	**OP787876**	**OP961937**	**This study**
*D. multiseptata*	MFLUCC 15-0609^T^	KX710140	KX710145	MF135659	[42]
*D. multiseptata*	MFLUCC 16-1044	MF077555	MF077544	MF135652	[2]
***D. nabanheensis***	**HJAUP C2003^T^**	**OP787877**	**OP787873**	**OP961935**	**This study**
*D. neorostrata*	MFLUCC 18-0376^T^	MN163017	MN163008	—	[22]
*D. nonrostrata*	KUNCC 21-10730^T^	OK341198	OK310699	OP413481	[21]
*D. obclavata*	MFLUCC 18-0329^T^	MN163010	MN163012	—	[22]
*D. obpyriformis*	DLUCC 0867	MG979765	MG979757	MG988423	[9]
*D. pachyconidia*	KUMCC 21-10724^T^	OK341194	OK310696	OP413477	[21]
*D. palmarum*	MFLUCC 18-1446^T^	MK079663	MK085062	MK087660	[3]
*D. phangngaensis*	MFLUCC 16-0857^T^	MF077556	MF077545	MF135653	[2]
*D. rayongensis*	MFLUCC 18-0415^T^	MH457137	MH457172	MH463253	[28]
*D. rostrata*	MFLUCC 16-0969^T^	MG979766	MG979758	MG988424	[9]
*D. rostrata*	DLUCC 0885	MG979767	MG979759	MG988425	[9]
*D. saprophytica*	MFLUCC 18-1238^T^	MW287780	MW286506	MW396651	[7]
*D. septate*	GZCC 22-0078^T^	ON527947	ON527939	ON533683	[20]
*D. songkhlaensis*	MFLUCC 18-1234^T^	MW287755	MW286482	MW396642	[7]
***D. sinensis***	**HJAUP C2044^T^**	**OP787875**	**OP787878**	**OP961936**	**This study**
*D. suoluoensis*	MFLUCC 17-0224^T^	MF077557	MF077546	MF135654	[2]
*D. suoluoensis*	MFLUCC 17-1305	MF077558	MF077547	—	[2]
*D. tectonae*	MFLUCC 12-0291^T^	KX751713	KX751711	KX751710	[42]
*D. tectonae*	MFLUCC 16-0946	MG979768	MG979760	MG988426	[9]
*D. tectonigena*	MFLUCC 12-0292^T^	KX751714	KX751712	—	[42]
*D. thailandica*	MFLUCC 16-0270^T^	MH260292	MH275060	MH412767	[10]
*D. thysanolaenae*	KUN-HKAS 112,710	MW879524	MW723057	MW729783	[17]
*D. thysanolaenae*	KUN-HKAS 102247^T^	MK064091	MK045851	MK086031	[13]
*D. tropica*	GZCC 22-0076^T^	ON527943	ON527935	ON533679	[20]
*D. verrucosa*	GZCC 20-0434^T^	MZ868762	MZ868771	MZ892977	[11]
*D. wuzhishanensis*	GZCC 22-0077^T^	ON527946	ON527938	ON533682	[20]
*D. xishuangbannaensis*	KUMCC 17-0290^T^	MH260293	MH275061	MH412768	[10]
*D. yongxiuensis*	JAUCC 4725^T^	OK562394	OK562388	OK562406	[6]
*D. yunjushanensis*	JAUCC 4723^T^	OK562398	OK562392	OK562410	[6]
*D. yunnanensis*	MFLUCC 20-0153^T^	MW081546	MW081541	MW084995	[16]

Notes: The ex-type cultures are indicated using “^T^” after strain numbers; “—” stands for no sequence data in GenBank.

**Table 2:** Synopsis of morphological characteristics, habitat, host, and locality compared across *Distoseptispora* anamorph species.

Species	Conidiophores (μm)	Conida		Habitat	Host/Locality	References
		Size (µm)	Morphology			
*Distoseptispora adscendens*	20–50 × 7–10	110–375 × 14–20	Cylindrical to obclavate, rostrate, brown to dark brown, paler towards the apex, 16–62-distoseptate	Terrestrial	Rotten wood and dead branches of many woody plant species, China	[21,45]
*D. amniculi*	90–180 × 3–4.5	85–167 × 9–11.8	Obclavate, rostrate, olivaceous-brown, grayish-brown or mid-brown, sometimes with a secondary conidium, (7–)12–24-distoseptate	Freshwater	Unidentified submerged wood, Thailand	[11]
*D. appendiculata*	62–86 × 4.5–5.5	67–89 × 10–16	Obpyriform or obclavate, olivaceous or dark brown, with a gelatinous sheath around tip, 13–17-distoseptate	Freshwater	Unidentified submerged wood, Thailand	[22]
*D. aqualignicola*	90–190(–240) × 5–8	41–94(–104) × 10.5–12.5	Obclavate, rostrate, brown at the base, subhyaline to pale-brown at the apex, 4–8-euseptate	Freshwater	Unidentified submerged wood, China	[21]
*D. aquamyces*	(78–)91–198 × 4–7	30–95 × 7–12	Obclavate to obpyriform, mostly rostrate, pale-brown to brown at the base, subhyaline to pale-brown at the apex, 4–10-euseptate, verrucose	Freshwater	Unidentified submerged wood, China	[21]
*D. aquatica*	29–41 × 7–9	110–157 × 13.5–16.5	Obclavate, dark brown with bluish-green to malachite green tinge, paler towards the apex, 15–28-distoseptate	Freshwater	Unidentified submerged wood, China	[1]
*D. aquisubtropica*	16–83 × 5–11	43–278 × 11–19	Obclavate or lanceolate, rostrate, pale brown or dark brown, olivaceous,16–31-distoseptate, verrucose	Freshwater	Unidentified submerged wood, China	[20]
*D. atroviridis*	100–167 × 2.7–4	31–43 × 13–20	Ellipsoidal to obovoid, dark green, subhyaline at the basal cell, 6-distoseptate	Freshwater	Unidentified submerged wood, China	[11]
*D. bambusae*	40–96 × 4–5.5	45–74 × 5.5–9.5	Obclavate, olivaceous or brown, 5–10-distoseptate	Terrestrial	Dead bamboo culms, China	[15]
*D.* *bambusicola*	64–116 × 4–7	72–193 × 7.5–14.5	Obclavate or lanceolate, rostrate, pale brown, up to 16-distoseptate	Freshwater	Unidentified submerged decaying bamboo culms, China	[18]
*D. bangkokensis*	37–55 × 3–4	400–568 × 13–16	Obclavate, rostrate, dark olivaceous to dark brown, conidia percurrent proliferation, which forms another conidium at the apex, multi-distoseptate	Freshwater	Unidentified submerged wood, Thailand	[17]
*D. cangshanensis*	44–68 × 4–8	58–166(–287) × 10–14	Obclavate or lanceolate, rostrate, olivaceous or brown, multi-distoseptate	Freshwater	Unidentified submerged wood, China	[9]
*D. caricis*	35–90 × 6–7	(55–)65–85(–100) × 15–16(–17)	Obclavate, brown, septa with central pore, basal cell pale brown, 5–10-distoseptate	Terrestrial	Leaves of *Carex* sp., Thailand	[26]
*D. chinensis*	16.5–44 × 5.5–9	81–283 × 10–19	Obclavate or lanceolate, rostrate, olivaceous to dark brown, up to 40-distoseptate	Freshwater	Unidentified submerged wood, China	[12]
*D. clematidis*	22–40 × 4–10	120–210 × 12–20	Oblong, obclavate, cylindrical or rostrate, brown with green tinge, lighter towards the apex, 28–35-distoseptate	Terrestrial	Dried stem of *Clematis sikkimensis*, Thailand	[41]
*D. crassispora*	14–27 × 6–10	95–197(–214) × 13–24	Obclavate, rostrate, brown with a green tinge, 15–36(–41)-distoseptate	Freshwater	Unidentified submerged wood, China	[21]
*D. curvularia*	11–28 × 5–9	(60–)100–200(–314) × 12–19	Obclavate, rostrate, brown with a green tinge, (9–)16–48(–59)-distoseptate	Freshwater	Unidentified submerged wood, China	[21]
*D. cylindricospora*	105–157 × 6.5–8.5	136.5–278 × 8.5–11	Cylindrical to elongated, greenish-brown to dark brown, hyaline at the apex, 20–65-distoseptate	Freshwater	Unidentified submerged wood, China	[19]
*D. dehongensis*	45–80 × 4–5	17–30 × 7.5–10	Obpyriform to obclavate, broad cylindrical or irregular, olivaceous, 3–5-distoseptate	Freshwater	Unidentified submerged wood, China	[3]
*D. effusa*	72–171 × 5–6.5	35.5–113 × 7–12.5	Obclavate, rostrate, olivaceous-brown to dark brown, sometimes slightly paler at the apex, 4–9-distoseptate	Freshwater	Unidentified submerged wood, China	[11]
*D. euseptata*	19–28 × 4–5	37–54 × 8–9	Obpyriform to obclavate, olivaceous, becoming paler towards the apex, 4–7-euseptate	Freshwater	Unidentified submerged wood, China	[16]
*D. fasciculata*	12–16 × 5–6	46–200 × 10–16.5	Subcylindrical to obclavate, olivaceous when young, dark brown when mature, 10–40-distoseptate	Freshwater	Unidentified submerged wood, Thailand	[7]
*D. fluminicola*	21–33 × 5.5–6.5	125–250 × 13–15	Oblong, obclavate, cylindrical or rostrate, brown with green tinge, paler towards the apex, 17–34-distoseptate	Freshwater	Unidentified submerged wood, China	[1]
*D. fusiformis*	40–110 × 3.5–5.8	35–52 × 13.5–22	Ellipsoidal to fusiform, dark olivaceous-brown to dark brown, pale brown at both ends, 6–8-distoseptate	Freshwater	Unidentified submerged wood, China	[11]
*D. guizhouensis*	21–50 × 4–9	90–273 × 15–21	Obclavate, brown to dark brown, usually paler towards apex, 10–38-distoseptate	Terrestrial	Unidentified decaying wood, China	[12]
*D. guttulata*	55–90(–145) × 3.5–5.5	75–130(–165) × 7–11	Obclavate or lanceolate, rostrate, mid- to dark brown or olivaceous, 11–14(–20)-euseptate	Freshwater	Unidentified submerged wood, Thailand	[2]
*D. hydei*	87–145 × 3–7	32–58 × 10–15	Obpyriform to fusiform, olivaceous to brown, with a hyaline, globose, gelatinous sheath around tip, 7–9-distoseptate	Terrestrial	Decaying bamboo culms, Thailand	[5]
*D. lancangjiangensis*	144–204 × 5–6	64–84 × 9–10	Narrowly obclavate or obspathulate, brown to dark brown, becoming paler or hyaline towards apex, 3–10-euseptate	Freshwater	Unidentified submerged wood, China	[17]
*D. leonensis*	110–130 × 6–8	50–75 × 15–18	Obclavate, fusiform or ellipsoidal, rostrate, medium brown, becoming pale brown towards the apex, 8–10-distoseptate	Terrestrial	Dead culms of grasses or dead branches, China	[21,45]
*D. lignicola*	84–124 × 4–5	60–108 × 7–9	Obclavate, solitary or catenate, brown, 5–9-euseptate	Freshwater	Unidentified submerged wood, Thailand	[22]
*D. longispora*	17–37 × 6–10	189–297 × 16–23	Obclavate, elongated, brown to yellowish-brown, 31–56-distoseptate	Freshwater	Unidentified submerged wood, China	[14]
*D. martinii*	50–110 × 3.5–4.5	15–20 × 11–16	Transversal ellipsoid, oblate or subglobose, muriform, pale brown to brown	Terrestrial	Unidentified dead branches, China	[8]
*D. meilingensis*	69–192 × 4–7	32–64.5 × (7–)9–12.5	Obclavate, mostly bright brown when mature, 5–7-distoseptate	Freshwater	Decaying bamboo culms, China	[6]
*D. mengsongensis*	17–54 × 4.5–7	86–200 × 6–13	Obclavate, brown to dark brown, sometimes with percurrent proliferation and forming another conidium from the conidial apex, 15–31-distoseptate	Terrestrial	Unidentified dead branches, China	This study
*D. multiseptata*	23–65 × 4.5–8.5	95–290 × 11–20	Obclavate, rostrate, dark olivaceous-green, multi-distoseptate	Freshwater	Unidentified submerged wood, Thailand	[42]
*D. nabanheensis*	29–42 × 8–10	102–214.5 × (7–)11–14.5	Obclavate, brown to dark brown, 18–31-distoseptate	Terrestrial	Unidentified dead branches, China	This study
*D. neorostrata*	93–117 × 5.5–6.5	109–147 × 13–15	Obclavate, rostrate, dark olivaceous to mid- or dark brown, pale brown towards apex, multi-distoseptate	Freshwater	Unidentified submerged wood, Thailand	[22]
*D. nonrostrata*	105–160 × 4.5–7	22–51 × 8–14	Oblong, obclavate or narrowly obpyriform, mostly non-rostrate, rarely rostrate, pale-olivaceous or pale-brown, 4–10-distoseptate	Freshwater	Unidentified submerged wood, China	[21]
*D. obclavata*	117.5–162.5 × 5–7	46–66 × 9–11	Obclavate, olivaceous to pale or dark brown, 9-11-distoseptate	Freshwater	Unidentified submerged wood, Thailand	[22]
*D. obpyriformis*	97–119 × 5–7	53–71 × 12–16	Obpyriform, olivaceous to pale or dark brown, 9–11-distoseptate	Freshwater	Unidentified submerged wood, China	[9]
*D. pachyconidia*	11–27 × 4–9	42–136 × 14–22	Obclavate, lanceolate, rostrate or not, pale-brown with a green tinge, 8–21-distoseptate	Freshwater	Unidentified submerged wood, China	[21]
*D. palmarum*	90–165 × 4–7	35–180 × 7–11	Oblong, obclavate, cylindrical or rostrate, greenish-black to brown, paler towards the apex, 7–27-distoseptate	Terrestrial	Rachis of *Cocos nucifera*, Thailand	[3]
*D. phangngaensis*	18–30(–40) × 4.3–6.5	165–350 × 14–19	Obclavate, rostrate, dark olivaceous to mid- or dark brown, multi-distoseptate	Freshwater	Unidentified submerged wood, Thailand	[2]
*D. rayongensis*	75–125 × 3.5–5.5	(36–)60–106(–120) × 9–14.5	Obclavate or obspathulate, rostrate, pale brown or pale olivaceous, becoming paler or hyaline towards the apex, sometimes with percurrent proliferation and forming another conidium from the conidial apex, mostly 9–13-euseptate, rarely 14–15-septate	Freshwater	Unidentified submerged wood, Thailand	[28]
*D. rostrata*	82–126 × 5–7	115–155 × 9–11	Obclavate or lanceolate, rostrate, olivaceous to pale brown, (15–)18–23-distoseptate	Freshwater	Unidentified submerged wood, China	[9]
*D. saprophytica*	50–140 × 3.2–4.2	14.5–30 × 4.5–7.5	Subcylindrical to obclavate, olivaceous to brown, solitary or occasionally catenate, 2–6-distoseptate	Freshwater	Unidentified submerged wood, Thailand	[7]
*D. septata*	23–86 × 3–7	22–179 × 10–16	Obclavate, rostrate, pale brown or dark brown, olivaceous-green, usually paler towards apex, verrucose, up to 25-distoseptate	Freshwater	Unidentified submerged wood, China	[20]
*D. sinensis*	23.5–56.5 × 3.5–7	40–107(–137) × 10–12	Obclavate, brown to dark brown, apical cell paler, 10–25-distoseptate	Terrestrial	Unidentified dead branches, China	This study
*D. songkhlaensis*	70–90 × 4–5.5	44–125 × 9–14.5	Obclavate, olivaceous to brown, 9–16-distoseptate	Freshwater	Unidentified submerged wood, Thailand	[7]
*D. suoluoensis*	80–250 × 4.5–5.8	(65–)80–125(–145) × 8–13	Narrowly obclavate or obspathulate, yellowish-brown or dark olivaceous, becoming paler or hyaline towards the apex, verrucose, sometimes with percurrent proliferation, which forms another conidium from the conidial apex, 8–10-euseptate	Freshwater	Unidentified submerged wood, China	[2]
*D. tectonae*	Up to 40 × 4–6	(90–)130–140(–170) × (11–)13–14(–16)	Cylindric-obclavate, dark reddish-brown, slightly paler towards the apex, verruculose, 20–28-distoseptate	Terrestrial	Dead twig of *Tectona grandis*, Thailand	[42]
*D. tectonigena*	Up to 110 × 5–11	(83–)148–225(–360) × (10–)11–12(–13)	Cylindric-obclavate, dark reddish-brown and slightly paler towards the apex, verruculose, 20–46-distoseptate	Terrestrial	Dead twig of *Tectona grandis*, Thailand	[42]
*D. thailandica*	15–26 × 3–6	130–230 × 13.5–17	Oblong, obclavate, cylindrical or rostrate, reddish-brown to brown, pale brown towards the apex, 35–52-distoseptate	Terrestrial	Dead leaves of *Pandanus* sp., Thailand	[10]
*D. thysanolaenae*	30–80 × 3.5–5.5	21.5–80 × 6.5–12.8	Narrow and elongated obclavate, light to dark brown, paler at the apex, 8–14-distoseptate	Terrestrial	Dead culms of *Thysanolaena maxima*, China	[13]
*D. tropica*	60–151 × 3.5–7	39–75 × 7.5–10.5	Obclavate, rostrate, olivaceous-brown or dark brown, verrucose, 5–7 distoseptate	Terrestrial	Unidentified dead wood, China	[20]
*D. verrucosa*	92–250 × 4.7–6.3	41–63 × 8.8–12.6	Obclavate, rostrate, olivaceous-brown, becoming paler at the apex, verrucose, 6–8-euseptate	Freshwater	Unidentified submerged wood, China	[11]
*D. wuzhishanensis*	16–56 × 5–7	76–143 × 11–17	Obclavate, rostrate, pale brown or dark brown, olivaceous-green and yellow, usually paler towards apex, verrucose, up to 22-distoseptate	Freshwater	Unidentified submerged wood, China	[20]
*D. xishuangbannaensis*	12–17 × 2–5	160–305 × 8–15	Cylindrical-obclavate, green brown to brown, up to 40-distoseptate	Terrestrial	Decaying leaves of *Pandanus utilis*, China	[10]
*D. yongxiuensis*	112–253 × 4–9	46–74(–86) × 10–13(–16)	Obclavate or obspathulate, olivaceous to yellowish-brown or brown, becoming paler or hyaline towards the apex, 6–9-euseptate	Freshwater	Decaying bamboo culms, China	[6]
*D. yunjushanensis*	100–175 × 5.5–10	39–67.5(–77) × (7–)9.5–13.5(–16.5)	Obpyriform or obclavate, olivaceous when young, dark brown when mature, sometimes with the percurrent proliferation which forms another conidium from the conidial apex, 7–13-distoseptate	Freshwater	Decaying bamboo culms, China	[6]
*D. yunnanensis*	131–175 × 6–7	58–108 × 8–10	Obclavate, rostrate, mid-olivaceous to brown, becoming paler towards the apex, 6–10-euseptate	Freshwater	Unidentified submerged wood, China	[16]

Notes: All conidia are smooth except where indicated.

**Table 3:** Synopsis of morphological characteristics, habitat, host, and locality compared across *Distoseptispora* teleomorph species.

Species	Asci (μm)	Ascospores			Habitat	Host/Locality	References
		Size (μm)	Septation	Characteristics			
*Distoseptispora hyalina*	145–190 × 8–11	(19.5–)23–26(–28.5) × 4.5–7	0–3	Fusiform, hyaline, guttulate, and with a mucilaginous sheath	Freshwater	Unidentified submerged wood, Thailand	[11]
*D. licualae*	120–210 × 8–15	20–30 × 5–10	0	Inequilateral to fusiform, hyaline, guttulate, and with a thick mucilaginous sheath	Terrestrial	Dead leaves of *Licuala glabra*, Thailand	[43]

## Data Availability

All sequences generated in this study were submitted to GenBank.

## Supplementary Materials

The following supporting information can be downloaded at: https://www.mdpi.com/article/10.3390/jof9040470/s1.
